# Inflammasome Contribution to the Activation of Th1, Th2, and Th17 Immune Responses

**DOI:** 10.3389/fmicb.2022.851835

**Published:** 2022-03-17

**Authors:** Ekaterina Martynova, Albert Rizvanov, Richard A. Urbanowicz, Svetlana Khaiboullina

**Affiliations:** ^1^Kazan Federal University, Kazan, Russia; ^2^Department of Infection Biology and Microbiomes, Institute of Infection, Veterinary and Ecological Sciences, University of Liverpool, Liverpool, United Kingdom

**Keywords:** inflammasome, Th1, Th2, Th17, immune response

## Abstract

Inflammasomes are cytosolic polyprotein complexes formed in response to various external and internal stimuli, including viral and bacterial antigens. The main product of the inflammasome is active caspase 1 which proteolytically cleaves, releasing functional interleukin-1 beta (IL-1β) and interleukin-18 (IL-18). These cytokines play a central role in shaping immune response to pathogens. In this review, we will focus on the mechanisms of inflammasome activation, as well as their role in development of Th1, Th2, and Th17 lymphocytes. The contribution of cytokines IL-1β, IL-18, and IL-33, products of activated inflammasomes, are summarized. Additionally, the role of cytokines released from tissue cells in promoting differentiation of lymphocyte populations is discussed.

## Introduction

In the course of evolution, two complementary systems aimed to detect and eliminate pathogens have developed: the innate and adaptive. The innate system is primed to react early after infection by recognition of pathogen-associated molecular patterns (PAMPs), a conserved molecular configuration derived from microorganisms and recognized as foreign by the receptors of the innate immune system (Silva-Gomes et al., 2014). The wide range of PAMPs are detected through a limited number of germline receptors named pattern recognition receptors (PRRs; Medzhitov and Janeway, 2002). These receptors are expressed by many cell types, including macrophages, monocytes, dendritic cells, neutrophils, and epithelial cells (Li and Wu, 2021).

PRRs are located on the cell surface as well as in the cytosol. Toll-like receptors (TLRs) are PRRs detecting external and internalized PAMPs (El-Zayat et al., 2019). These receptors recognize microbial components (Gram-positive and Gram-negative bacteria, mycobacteria, RNA and DNA viruses, and fungi) at an early stage of the immune response (McDonald et al., 2005). There are also PRRs located in the cytosol: NOD-like receptors (NLR) and RIG-1-like receptors (RLR; Evavold and Kagan, 2018). NOD-like receptors are only able to recognize bacterial structures, while RLRs recognize viral components (Girardin et al., 2003; McDonald et al., 2005; Kawai and Akira, 2009; Li et al., 2014). As well as TLRs, some NLRs, in particular NOD1 and NOD2, can downstream activate nuclear factor NF-kB, a key transcription factor in inflammation (Hasegawa et al., 2008).

In addition to PAMPs, damage-associated molecular patterns (DAMPs) released by injured tissue (Moriyama and Nishida, 2021) can also activate inflammasomes. Multiple DAMPs are identified example are histones, DNA, ATP, reactive oxygen radicals (ROS), heat-shock proteins, and uric acid crystals (Abderrazak et al., 2015). On release from damaged cells, they can trigger non-microbial inflammation (Chen and Nuñez, 2010) initiated by an activated inflammasome. DAMPs have various structures; therefore, it could be suggested that they are less likely to directly bind an inflammasome. Instead, DAMPs use different mechanisms to activate inflammasomes. The initial step is recognition of DAMPs by TLRs and NLRs (Chen and Nuñez, 2010) that trigger various downstream pathways. One of these is potassium ion efflux (Gong et al., 2018). Potassium efflux can be induced by toxins, such as nigericin (Katsnelson et al., 2015). Additionally, P2X purinoceptor 7 (P2X7), pannexin-1, and K2P channels could contribute to the release of potassium ions into the extracellular space (Hafner-Bratkovič and Pelegrín, 2018; Xu et al., 2020). Potassium efflux could also be a part of the more complex response to DAMPs, as was shown by Li--Weber and Krammer (2003). In this study, ROS release by mitochondria and lysosomal membrane permeabilization was demonstrated after exposure to nigericin, a bacterial toxin causing potassium ion efflux. This decreased intracellular level of potassium ions could lead to conformational changes in inflammasome molecules required for its activation (Xu et al., 2020).

Like some TLRs, RLRs recognize viral nucleic acid and activate several proteins of the interferon regulatory family (IRF; Månsson Kvarnhammar et al., 2013). Interestingly, there is evidence of cross-talk between PRRs, as the specific function of NLRs depends on the initial activation of TLR signaling (Askarian et al., 2018). For example, NACHT leucine-rich repeat protein (NLRP) 1, NLRP3, and NLRC4 (formerly known as Ipaf) recognize bacterial components and activate caspase-1 (Cas1), a key inflammatory caspase that releases pro-inflammatory cytokines IL-1β and IL-18. TLRs, as well as NOD1 and NOD2, can activate the synthesis of IL-18 and IL-1β precursors by stimulating NF-kB (O’Neill, 2003).

For the production of IL-1β and IL-18, TLRs recruit a complex protein known as the inflammasome. The term inflammasome was introduced by Martinon et al. (2002) to describe a high molecular weight complex that activates Cas1. The inflammasome consists of a central protein, which, in its active form, recruits an apoptosis-associated speck-like protein containing a CARD (ASC). CARD of ASC then recruits pro-caspase1, which cleaves pro-IL-1β and pro-IL-18 releasing active cytokines, essential mediators of inflammation (Sun and Scott, 2016). In addition to inflammation, inflammasomes are linked to the mechanism of Cas1 activated cell death named pyroptosis (Heymann et al., 2015).

Inflammasomes are activated by PAMPs and DAMPs during natural infection and artificially induced immune responses by vaccination (Crooke et al., 2021). Multiple studies have demonstrated that activating the inflammasome could serve as an adjuvant potentiating immune response (Li et al., 2007; Bueter et al., 2011; Marty-Roix et al., 2016; Russell, 2016; Ivanov et al., 2020). These data suggest that targeting inflammasomes by adjuvants is a potential mechanism of enhancing the immune response (Ivanov et al., 2020). This enhancement of the immune response is often linked to the release of IL-1β and IL-18 (Li et al., 2007; Bueter et al., 2011), establishing local inflammation and attracting leukocytes. However, it appears that inflammasomes could contribute to the development of the adaptive immune response, as a lack of ASC protein was linked to complete ablation of antigen-specific CD4^+^ T-cell proliferation and failure to develop a specific immune response (Seydoux et al., 2018). A growing body of evidence substantiates the notion that inflammasomes could contribute to the formation of a specific immune response. In this review, we summarize evidence of the inflammasome contribution to the T helper 1 (Th1), Th2, and Th17 immune response. The mechanisms of the inflammasome affecting Th cell differentiation are also discussed.

## Inflammasome Structure

**Figure 1:**
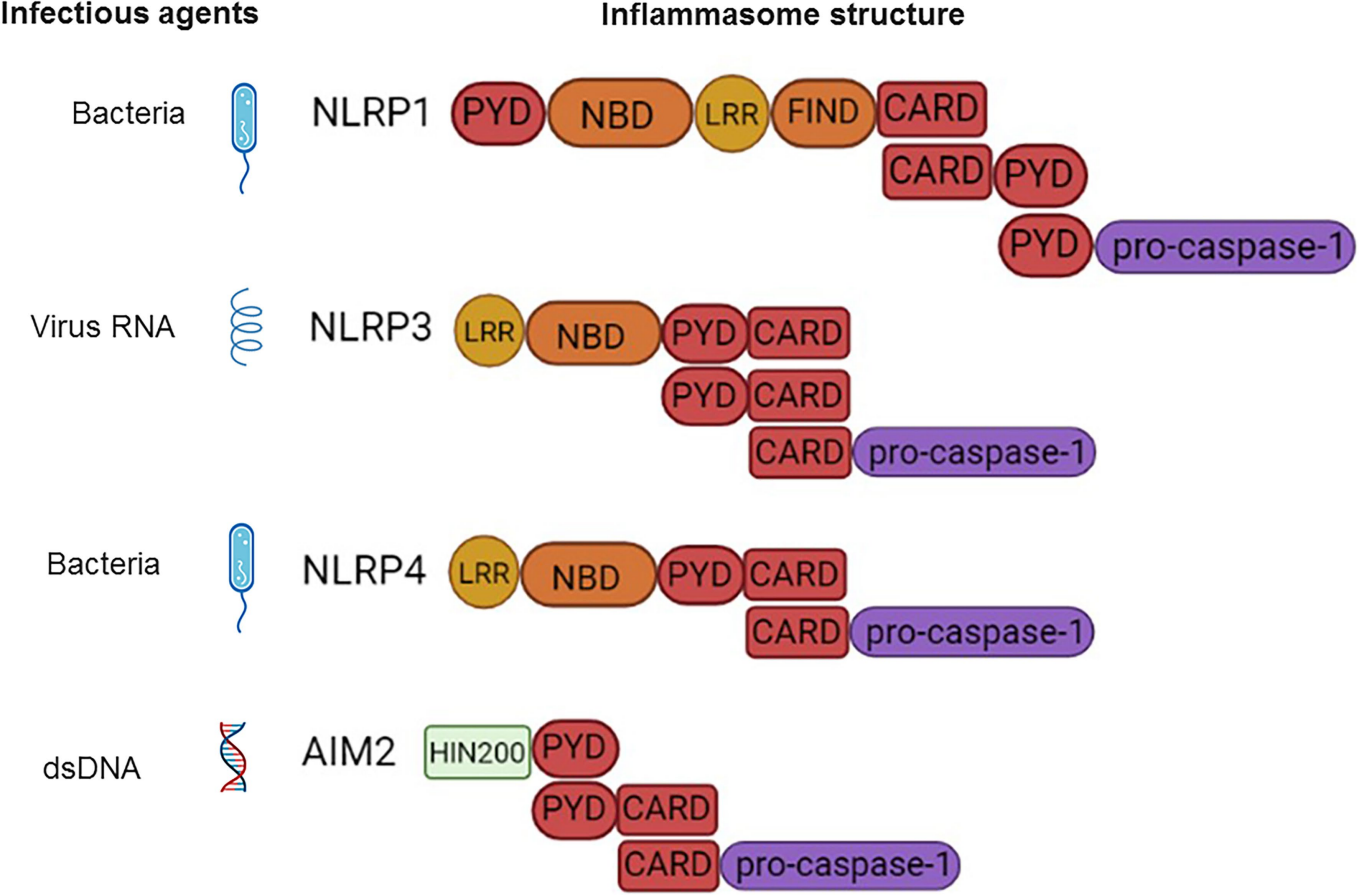
Schematic presentation of the inflammasome structure and pathogen-associated molecular patterns (PAMPs) ligands. NLRP1-CARD binds to and forms a dimer with CARD of pro-caspase-1. NLRP3-CARD-PYD polymerizes with adaptor ASC (PYD-CARD). This is followed by CARD dimerization with CARD-pro-caspase-1. NLRC4-CARD forms a dimer with CARD of ASC (PYD-CARD) adaptor. The PYD of ASC then polymerizes with PYD of the second ASC adaptor molecule. This second ASC CARD binds to CARD of pro-caspase-1. AIM2-PYD dimerizes with PYD of ASC (PYD-CARD), followed by CARD of ASC binding to CARD-pro-caspase-1.

The canonical inflammasome response is associated by engagement of two classes of cytosolic receptors: nucleotide-binding and oligomerization domain (**N**ODs) **L**ike **R**eceptors (NLRs) and absent in melanoma 2 (**A**IM2) **L**ike **R**eceptors (ALRs). There are 23 NLRs encoded in the human genome (Harton et al., 2002), however, only **NLR** family **P**yrin domain-containing 1 (NLRP1), NLRP3, NLRP6, NLRP7, NLRP12, and NLR family CARD domain-containing protein 4 (NLRC4) can form inflammasome complexes (Shenoy et al., 2012). The NLR structure is characterized by a central NOD, which are flanked by a C-terminal leucine-rich repeat (LRR) and a N-terminal CARD pyrin domain (PYD; Schroder and Tschopp, 2010; Zheng et al., 2020; Figure 1). In all NLRs (except NLRP10), LRR mediates ligand binding, while CARD or PYD function for protein–protein interaction (Li et al., 2007; Russell, 2016). AIM2 contains a N-terminal PYD and C-terminal **H**ematopoietic **I**nterferon-inducible **N**uclear (HIN) protein domain and it can also form an inflammasome (Bueter et al., 2011). The unifying feature of inflammasome forming-receptors is the presence of the pyrin domain (PYD) or CARD.

**Figure 2:**
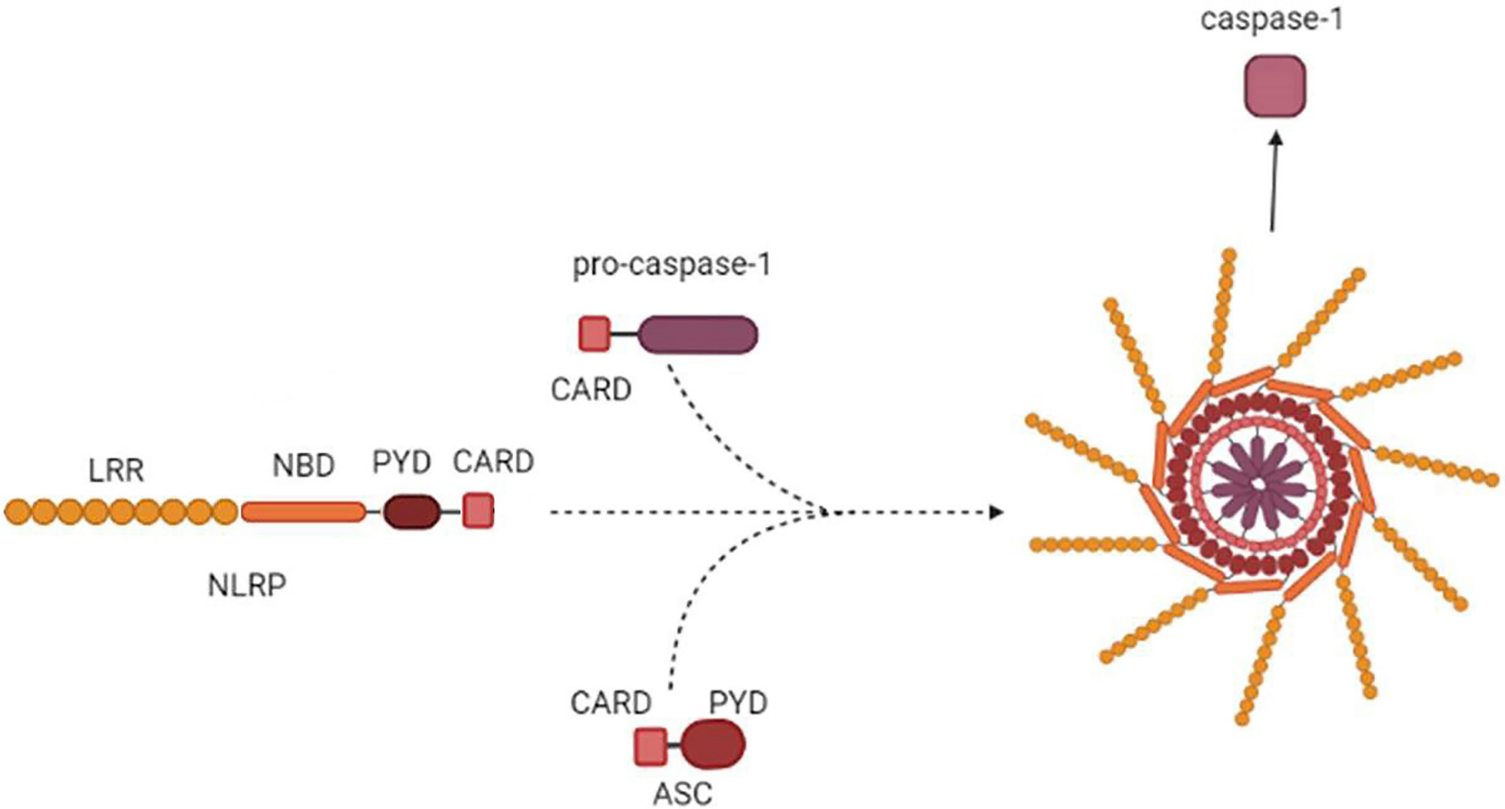
Inflammasome assembly. NLRP3 is used as an example to demonstrate inflammasome assembly. NALRP3 PYD-CARD polymerizes with PYD-CARD of an ASC adaptor. The adaptor CARD then forms a dimer with CARD of pro-caspase-1. The macromolecule formed by oligomerized NLRP3-ASC-pro-caspase-1 complex is assembled into the multi-subunit, wheel-shaped inflammasome complex, which releases active Cas1.

Inflammasome formation is initiated by sensing the endogenous or exogenous stimuli (LRR) followed by coordinated assembly of the platform including sensors (NAIP/NLRC4, NLRP3/6/7, AIM2), adapters (ASC), and downstream effectors (Figure 2; Seydoux et al., 2018).

As the result, the multiprotein proteolytic complex is formed, recruiting pro-caspase-1. The role of this complex is to liberate Cas1, which becomes active and cleaves pro-IL-1β and pro-IL-18 releasing active cytokines (Harton et al., 2002; Ting et al., 2008; Schroder and Tschopp, 2010; Shenoy et al., 2012; Zheng et al., 2020). In addition to the release of cytokines, inflammasome activation is accompanied by a specific form of cell death called pyroptosis (Wang et al., 2020). Pyroptosis is characterized by Cas1 cleavage of the gasdermin D (GSDMD) protein (Broz, 2015), which leads to cell lysis and subsequent release of cytoplasmic components, including mature forms of IL-1β and IL-18 (Sansonetti et al., 2000).

## Inflammasome Activation Mechanisms

### NLRP1 Inflammasome

NLRP1 was discovered as one of the first in the class of proteins that form inflammasomes (Kostura et al., 1989). It is predominantly expressed by epithelial cells and hematopoietic cells (Broz, 2015). This inflammasome is characterized by the activation of pro-inflammatory proteases, including Cas1 (Rathinam et al., 2012). In turn, Cas1 releases IL-1β and IL-18 (Ghayur et al., 1997), as well as the pore-forming GSDMD protein (Zheng et al., 2020). GSDMD makes pores in the cell membrane leading to a pyroptosis (Gu et al., 1997). The presence of an unusual function-to-find domain (FIIND) is unique for NLRP1 (Fink and Cookson, 2006). This domain has protease activity, which can cleave inactive NLRP1 releasing the bioactive CARD domain. The self-activating activity of NLRP1 is linked to the pathogenesis in vitiligo, rheumatoid arthritis, and Crohn disease (Bertin et al., 1999; Fink and Cookson, 2005; Kummer et al., 2007). In addition to self-activation, studies using a murine model demonstrated that NLRP1 can be activated by microbial antigens, such as the lethal protease factor *Bacillus anthracis* (Broz and Dixit, 2016) and E3-ubiquitin ligases from *Shigella flexneri* (Shaftel et al., 2008). Based on data collected, a model of functional degradation was proposed to explain the mechanism of NLRP1 activation (Shaftel et al., 2008). This model suggests that the degradation of the N-terminal domains by microbial proteasomes liberates the C-terminal fragment which is a potent Cas1 activator (Shaftel et al., 2008).

In humans, NLRP1 directly binds to muramyl dipeptide (MDP), which leads to conformational changes in the structure of NLRP1, thereby activating ATP (Sandall et al., 2020). ATP hydrolysis induces oligomerization of NLRP1 and activates Cas1. Interestingly, MDP-mediated activation of NLRP1 Cas1 does not require the involvement of the ASC protein. Together with Cas1, caspase 5 is also involved in the binding of the NLRP1 complex (Martinon et al., 2002).

NLRP1 activation was shown by the lethal factor of *B. anthracis* (Chavarría-Smith and Vance, 2013) and ubiquitylation by *S. flexneri* effector IpaH7.8 (Sandstrom et al., 2019). The lethal factor liberates the C-terminal end of NLRP1 containing FIIND-CARD fragment, that can recruit Cas1 and initiate the inflammasome assembly (Chui et al., 2019). NLRP1 can also be activated by intracellular pathogens indirectly by sensing the metabolic stress as was shown for *Listeria monocytogenes* and *S. flexneri* (Neiman-Zenevich et al., 2017). Similarly, indirect activation of NLRP1 was suggested by Ewald et al. (2014) in cells infected with *Toxoplasma gondii*. This activation appears to have a distinct mechanism independent of the lethal factor. It appears that NLRP1 activation has a protective effect as it limits the parasite load and prevents dissemination. In contrast, Tye et al. (2018) have shown that inhibition of NLRP1 prevents colitis and increases the population of butyrate-producing commensals from the Clostridiales order. Also, acute lung injury was demonstrated in mice exposed to the *B. anthracis* lethal factor (Kovarova et al., 2012). Tissue injury was explained by activation of pyroptosis due to the NLRP1 activation.

NLRP1 activation has also been demonstrated in virus-infected cells. Recently, it was shown that the picornavirus encoded 3C protease, termed 3C^pro^ (Sun et al., 2016), could cleave and activate this inflammasome (Robinson et al., 2020). Following this observation, Tsu et al. (2021) have demonstrated that this protease, from multiple genera of viruses, could activate NLRP1 in multiple sites. In contrast, Kaposi sarcoma herpes virus (KSHV) open reading frame 63 (Orf63) was identified as a viral homolog of NLRP1 (Gregory et al., 2011). Orf63 could block NLRP1-dependent cas1 activation as well as IL-1β and IL-18 processing. It appears that the inhibition of NLRP1 is necessary for reactivation and virus replication.

### NLRP3 Inflammasome

Among all inflammasomes activated by NOD-like receptors, the NLRP3 is the most studied (Martinon et al., 2002). Unlike other sensory proteins, NLRP3 can respond to various non-pathogenic factors, environmental as well as endogenous. These factors could lead to aberrant activation of the NLRP3 inflammasome, which has been linked to the development of such complex pathologies as type 2 diabetes, atherosclerosis, gout, and neurodegenerative diseases (Broderick et al., 2015).

The NLRP3 inflammasome contains three distinct domains: the N-terminal PYD mediating homotypic binding; the nucleotide binding and oligomerizing domain (NACHT), which mediates ATP-dependent oligomerization; and the C-terminal LRR, which recognizes the ligand. While other NLR receptors also have a CARD as part of the primary sequence, NLRP3 requires an adapter protein to bind to pro-caspase1. The adapter is the ASC protein containing the CARD, which consists of PYD and CARD (Inouye et al., 2018).

It is believed that activation of the NLRP3 inflammasome in macrophages, dendritic cells, and microglia cells requires two signals. The first signal is called priming, which is usually induced by a TLR ligand, such as lipopolysaccharide (LPS; Bauernfeind et al., 2009). The role of priming is to increase the expression of the NLRP3 as well as the Cas1 substrate pro-IL-1β genes, which are normally present at low levels (Bauernfeind et al., 2009). The second signal triggers the assembly and activation of the inflammasome. This signal can be caused by bacterial toxins (Lee et al., 2015), ionophores (Mariathasan et al., 2006; Kasper et al., 2018), uric acid (Martinon et al., 2006), and bacterial RNA (Eigenbrod and Dalpke, 2015).

Similar to NLRP1, NLRP3 recruits GSDMD, where it could be split by Cas1 releasing C and N termini (He et al., 2015). The cleaved N terminus can auto-oligomerize upon interaction with phosphoinositides, embed into the membrane, and form a large circular pore (Liu et al., 2016). N terminus oligomers are mainly located on the inner leaflet of the membrane, killing from within the cell, without harming the neighboring cells when it is released during pyroptosis (Liu et al., 2016).

As wide array of agonists activating the NLRP3 inflammasome have been identified, many of which are PAMPs. Microbial activators are found among both Gram-positive and Gram-negative bacteria: *Staphylococcus aureus*, *L. monocytogenes*, *Streptococcus pneumonia*, and *Neisseria gonorrhoeae* (Vladimer et al., 2013). Also, fungi, *Candida albicans*, *Aspergillus fumigatus*, and *Microsporum canis*, as well as parasites, *Plasmodium chabaudi*, *Leishmania amazonensis*, and *Schistosoma mansoni*, were shown to activate NLRP3 (Joly and Sutterwala, 2010; Clay et al., 2014). Additionally, NLRP3 activation was shown by infection with RNA and DNA viruses, such as influenza virus, adenovirus and respiratory syncytial virus (Lupfer and Kanneganti, 2013). Having structurally diverse ligands derived from various infectious agents, Ulland et al. (2015) have suggested that NLRP3 activation is induced by cellular stress signal signals generated in infected cells. These signals are released during the rupture of lysosomes, stimulation of P2X7R, potassium ions efflux, and mitochondrial ROS (Kim and Jo, 2013). The potassium efflux was demonstrated in group B Streptococcus (GBS) infection as a mechanism of NLRP3 activation, where the β-hemolysin, the major virulence factor, was shown to play the central role (Costa et al., 2012). It appears that NLRP3 is protective, as mice lacking NLRP3 were more susceptible to infection as compared to wild-type (wt) animals. Similar mechanism of NLRP3 activation was demonstrated during *S. aureus* and *Streptococcus pneumoniae* infection, where α-hemolysin and pneumolysin were identified as playing the central role, respectively (Kebaier et al., 2012; Miller et al., 2020). Like in GBS infection, NLRP3 activation was protective, reducing animal’s susceptibility to infection with *S. aureus*. The mitochondrial dysfunction and apoptosis were suggested as contributing to NLRP3 activation in *Chlamydia pneumoniae* infection (Shimada et al., 2012). The release of oxidized mitochondrial DNA into the cytosol triggered the apoptotic signals activating NLRP3. It was suggested that it is the mitochondrial dysfunction that initiates the ROS release, intracellular K^+^ and lysosomal degradation, a diverse range of signals for NLRP3.

### NLRC4 Inflammasome

NLRC4 was originally described by Poyet et al. (2001) as a pro-apoptotic protein. Authors were searching for structural homologues of the apoptosis protease-activating factor 1 (APAF1) protein, which activates apoptotic caspases in response to cytosolic cytochrome C, and also contains the CARD domain and ATP binding sites. As a result, they discovered the IL-1β-converting enzyme (ICE)-protease-activating factor (IPAF), also known as (NLRC4; Sutterwala and Flavell, 2009), an enzyme capable of activating Cas1 (Poyet et al., 2001).

The assembly of the NLRC4 inflammasome complex occurs upon recognition of flagellated or type 3 secretion system (T3SS) expressing bacterial pathogens (Mariathasan et al., 2004; Franchi et al., 2006; Zamboni et al., 2006). Activation of NLRC4 requires neuronal apoptosis inhibitory protein (NAIP) sensing the bacterial flagellin or T3SS (Kofoed and Vance, 2011; Zhao et al., 2011). Using cryo-electron microscopy, Zhang et al. (2015) were able to reconstruct the structure of this inflammasome. The structural changes in activated NAIPs initiate the recruitment and oligomerization of NLRC4, which function as an adaptor to recruit and activate Cas1 (Hu et al., 2015). Once active Cas1 is released, it will cleave pro-IL-1β and pro-IL-18 as well as GSDMD (Lamkanfi and Dixit, 2014). Cytokines and GSDMD led to inflammation pyroptosis, similar to NLRP1 and NLRP3 activation.

Various Gram-negative bacteria belonging to the genera *Pseudomonas*, *Salmonella*, and *Yersinia* are shown to activate NLRC4 (Miao et al., 2008; Brodsky et al., 2010; Wick, 2011). Specifically, the type 3 and 4 secretion system (TT3S/TT4S) as well as flagellin were shown to activate this inflammasome (Miao et al., 2008; Zhao et al., 2011). The main function of the secretion system proteins is to form holes in the cell membrane permitting the entry of the microbial virulence factors (Galán and Wolf-Watz, 2006). NLRC4 activation by *Salmonella typhimurium*, *Legionella pneumophila*, and *Pseudomonas aeruginosa* appears to be possible by cytoplasmic transmission of flagellin (Franchi et al., 2006; Miao et al., 2006; Lightfield et al., 2008). These data suggest that NLRC4 is activated by TT3S, TT4S, or flagellin. Several members of the NLR family Apoptosis Inhibitory Proteins (NAIPs) family have been implicated in activating the inflammasome (Lightfield et al., 2008; Kofoed and Vance, 2011). Studies have suggested that NAIPs could serve as a ligand-sensing activator of NLRC4 (Rayamajhi et al., 2013; Rauch et al., 2016). There is a single human NAIP (hNAIP; Endrizzi et al., 2000) that detects T3SS and flagellin (Rayamajhi et al., 2013; Kortmann et al., 2015).

## Inflammasome-Independent PROCESSING of Pro-IL-1β and Pro-IL-18

Cas1 is a key protease in the processing of pro-IL-1β and pro-IL-18 (Afonina et al., 2015). However, studies using a mouse model revealed that in addition to cas1, cas11 could cleave pro-IL-1β, although with lower efficacy (Kang et al., 2000). There is growing evidence of cas8 involvement in cas1-independent pro-IL-1β processing. There are several mechanisms identified to explain cas8 cleavage of this cytokine. One of them is described as a macrophage and dendritic cells reaction to the stress, where TLR3 or TLR4 stimulation initiates the downstream activation of cas8 required for active IL-1β release (Antonopoulos et al., 2013; Shenderov et al., 2014). Interestingly, it appears that the cas8 could serve as NLRP3 effector in the absence of cas1 to process pro-IL-1β (Antonopoulos et al., 2015). In addition to TLRs, ligand binding to dectin-1 or Fas receptors could activate cas8 and initiate cas1-independent maturation of pro-IL-1β and pro-IL-18 (Bossaller et al., 2012; Gringhuis et al., 2012).

In addition to cas1, 8, and 11, other proteases could cleave pro-IL-1β. Elastase and cathepsin G, proteases found in neutrophils, could process pro-IL-1β (Korkmaz et al., 2010; Mankan and Hornung, 2013; Mizushina et al., 2019). Interestingly, elastase could be released by neutrophils and initiate cytokine processing in the extracellular space (Clancy et al., 2018). As a result, neutrophil elastase could promote IL-1β secretion by neighboring cells (Alfaidi et al., 2015). Another enzyme, proteinase 3, was also shown to contribute to pro-IL-1β and pro-IL-18 processing (Coeshott et al., 1999; Sugawara et al., 2001).

Some non-inflammasome dependent mechanisms of IL-18 and IL-1β activation overlap and are shown to process pro-IL-18. Granzyme B-mediated activation of IL-18 as well as increased IL-18 release upon incubation of keratinocytes with CD8^+^ T cells was demonstrated (Akeda et al., 2014; Wensink et al., 2015). These data could be evidence of a positive feedback loop between CD8^+^ T lymphocytes and local cells to promote type T-cell response, which is supported by IL-18 (Nakanishi, 2018). The assumption that the Granzyme B could have non-cytotoxic activity and, instead, contribute to development of the local immune response is supported by Hernandez-Pigeon (Hernandez-Pigeon et al., 2006). Authors have shown that upon the ultraviolet B (UVB) light exposure, human keratinocytes produce Granzyme B. Also, release of Granzyme B to the extracellular matrix was demonstrated in keratinocytes exposed to UVA light (Hernandez-Pigeon et al., 2007).

## IL-1β and IL-18 and the Specific Immune Response (Th1, Th2, and Th17)

### Inflammasome and Specific Immune Response

In order to commit to a certain Th cell lineage, naive CD4^+^ T cells are influenced by cytokines and other cellular signals provided by the immediate milieu upon encounter with their cognate antigen. T cells then interpret these signals and differentiate into particular Th cell subsets (Saravia et al., 2019). Some of these cytokines, specifically, IL-1β and IL-18, are the products of an activated inflammasome. Also, IL-33 was shown to be processed by Cas1 (Martin et al., 2012) and contribute to the Th cells differentiation. It appears that IL-1β and IL-18 play a role as initiators of the specific immune response, triggering the release of an additional subset of cytokines from the tissue cells. It is through the release of IL-1β and IL-18, inflammasomes initiate inflammation and stimulate specific immune response. This combination of IL-1β, IL-18, and tissue-specific cytokines will direct Th differentiation. IL-1β and IL-18 could also serve as a bridge between the innate and acquired immune response by themselves stimulating Th1, Th2, and Th17 populations of lymphocytes (Bruchard et al., 2015).

IL-1β and IL-18 belong to the IL-1 cytokine family (Dinarello, 2018). Their biological characteristics are summarized in Table 1. IL-1 signaling is central in the mechanism of the adaptive immune response by stimulating leukocyte migration, differentiation, and recruitment (Garlanda et al., 2013; Heymann et al., 2015). IL-1β employs IL-1R for the signaling (Svenson et al., 1993) which can also upregulate IL-2R (CD25) surface expression (Plaetinck et al., 1990; Bartel, 2009). Increased expression of IL-2R provides survival and proliferation signals to primed, naïve T cells. IL-1β also serves as a proliferation and survival stimulus to effector/memory T cells while attenuating regulatory (Treg) lymphocytes (O’Sullivan et al., 2006). Also, IL-1β is required for differentiation of the IL-17 producing Th17 lymphocytes (Acosta-Rodriguez et al., 2007).

**Table 1:** IL-1β and IL-18 characteristics.

	IL-1β	IL-18	References
Receptor	IL-1R	IL-18Rα	Acosta-Rodriguez et al., 2007; Garlanda et al., 2013
Function	Pro-inflammatory	Pro-inflammatory	
IFN-γ Production	Yes	Yes	Okamura et al., 1995; Thomassen et al., 1998
Th1 Proliferation	Yes	Yes, combined with IL-12	Sutton et al., 2006; Couper et al., 2008
Th2 Proliferation	No	Yes, without IL-12	Anderson et al., 2007
Th17 Proliferation	Promotes Th17 commitment; combined with IL-6 and IL-23 promotes pathogenic Th17	No	Suzuki et al., 2001; Qin et al., 2009; O’Reilly et al., 2014

IL-18 signals through heterodimeric receptor complex consisting of IL-18Rα, having significant homology to IL-1R (Thomassen et al., 1998). Therefore, signaling pathways induced by IL-18 are similar to those used by IL-1. IL-18 was first identified as an interferon γ (IFN-γ) inducing cytokine, which is a potent inducer of Th1 differentiation (Okamura et al., 1995; Kohno et al., 1997). Combined with IL-12, IL-18 can increase IFN-γ by Th1 lymphocytes and natural killer (NK) cells (Ahn et al., 1997; Xu et al., 1998). As the result, IL-18 could promote development of a Th1 response.

These early data provide strong evidence that inflammasomes not only contribute to inflammation, but also help to promote differentiation of naïve Th0 CD4^+^ T cells into a Th1 and Th2 subsets.

### Inflammasome and the Th1 Immune Response

Th1 lymphocytes differentiate from naïve Th0 cells when exposed to antigen presented by antigen-presenting cells (APCs) together with T-cell receptor (TCR) and CD28 co-stimulatory molecule binding (Gutcher and Becher, 2007). To direct Th1 differentiation, APC should provide one more stimulus, such as IL-12 cytokine, which will support lymphocyte antigen-specific cells proliferation (Hamza et al., 2010). These Th1 cells are characterized by production of high levels of IFN-γ, IL-2, and TNFα (Swanson et al., 2001; Kisuya et al., 2019). Expression of the transcription factor T-bet, responsible for expression of IFN-γ, is found in committed Th1 effectors (Szabo et al., 2000). Factors favoring Th1 differentiation also include signal transducer and activator of transcription 1 (STAT1; Ma et al., 2010), STAT5 (Liao et al., 2011) and STAT4 (Nishikomori et al., 2002) signaling. Th1 lymphocytes support the cellular immune responses associated with differentiation of CD8^+^ T cells into cytotoxic T lymphocytes (CTLs) as well as their survival (Schüler et al., 1999; Huang et al., 2007). These CTLs can secrete the cytolytic mediators (perforin and granzymes) and induce apoptosis in target cells (Trapani and Smyth, 2002). Moreover, activated CTLs secrete IFN-γ and TNF-α, which could enhance antigen presentation and provide positive feedback to the Th1 lymphocyte population (Sasiain et al., 1998). Additionally, Th1 lymphocytes could activate phagocytosis (Khan et al., 2016), and production of complement-fixing antibodies (Hammers et al., 2011), thus playing an important role in protection against infectious pathogens. Th1 type immune responses have been shown to be protective against viral (Snell et al., 2016), fungal (Jarvis et al., 2013), and bacterial infections (D’Elios et al., 2011).

IL-1β release upon activation of NLRP3 in an APCs can support T-cell priming (Hatscher et al., 2021), including enhancement of IFN-γ and IL-17 release by CD4^+^ T cells (Sutton et al., 2006; Feriotti et al., 2017). In contrast to myeloid cells, IL-1β production in T lymphocytes is lower and it is tightly regulated by an autocrine C5aR1/C5aR2 activation balance (Arbore et al., 2016). It was suggested that control of local IL-1β production is critical for the prevention of excessive and prolonged stimulation of Th1 lymphocytes (Arbore et al., 2016). In contrast to IL-1β, IL-18 does not stimulate differentiation of Th1 lymphocytes directly; however, it can support the commitment of Th1 lymphocytes by inducing the release of IFN-γ (Okamura et al., 1995; van den Eeckhout et al., 2020). Interestingly, this IFN-γ could provide positive feedback potentiating IL-1β release by human monocytes and Th1 cells (Chizzolini et al., 1997; Masters et al., 2010). The role of the inflammasome in supporting the Th1 immune response was also demonstrated using an animal model (Gurung et al., 2015a; Alhallaf et al., 2018).

Both IL-1β and IL-18 employ IFN-γ in promoting a Th1 immune response. Once Th1 lymphocytes are activated they become one of the prime producers of IFN-γ (Tominaga et al., 2000), together with CTLs, γδ-T cells, and NK cells (Kasahara et al., 1983; Girardi et al., 2001; Yu et al., 2006; Matsushita et al., 2015). The additional release of this cytokine can establish a positive loop where IFN-γ produced by activated leukocytes will keep directing Th1 differentiation (Bradley et al., 1996). Th1 supporting role of IFN-γ also includes inhibition of Th2 cell differentiation (Oriss et al., 1997) and IL-4 production (Naka et al., 2001).

It appears that the duration of the inflammasome stimulation is critical for Th1 response activation. Gurung et al. (2015b) have demonstrated that, in contrast to acute stimulation, chronic LPS stimulation dampens NLRP3 activation. This is the result of the regulatory IL-10 cytokine release, which inhibits the inflammasome. Similar IL-10 caused inhibition of NLRP3 inflammasome was demonstrated by Greenhill et al. (2014). This could be a mechanism aimed to prevent chronic Th1 activation as IL-10 is a potent inhibitor of the Th1 immune response (Couper et al., 2008).

**Figure 3:**
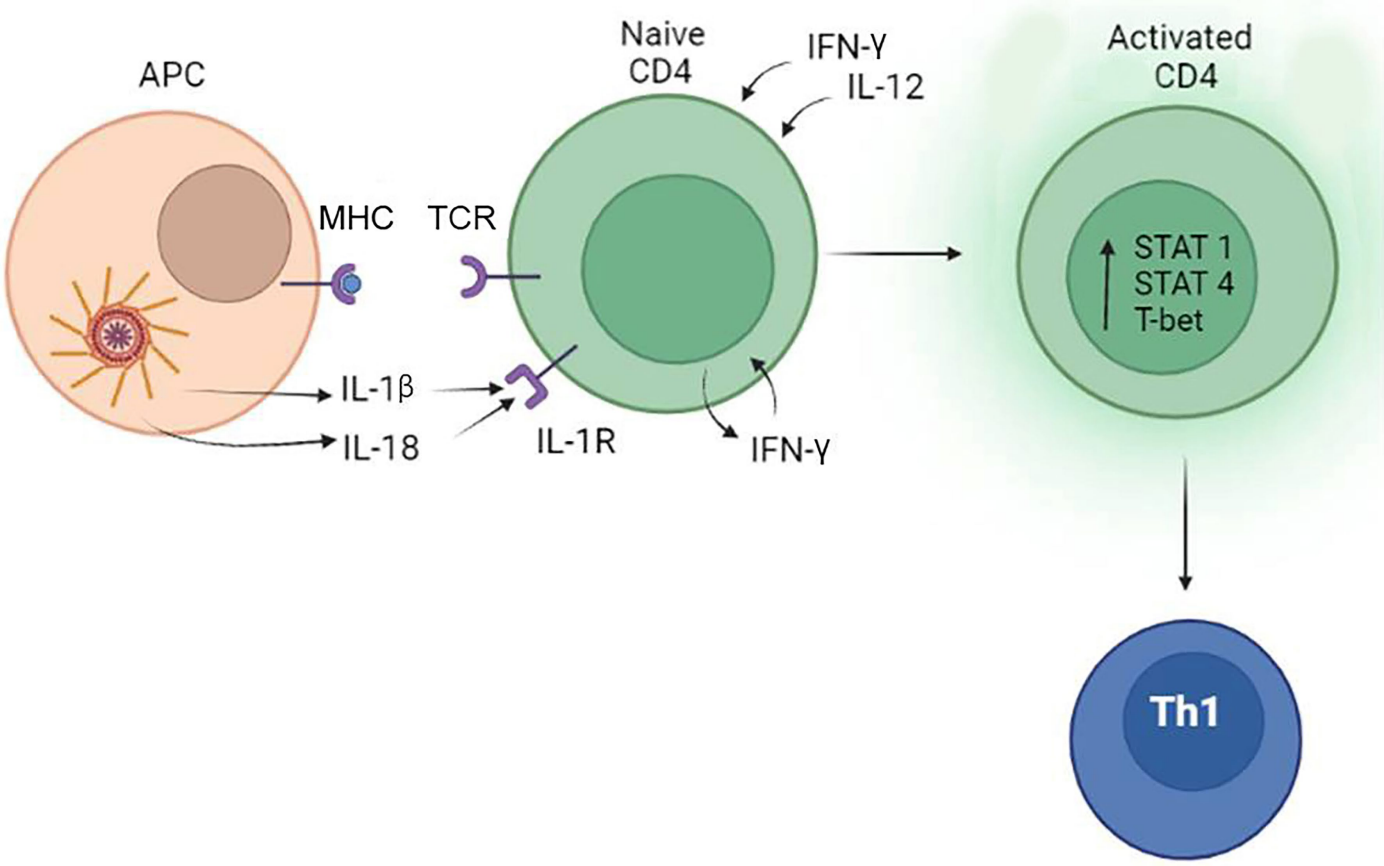
The role of the inflammasome in the differentiation of Th1 lymphocytes. Inflammasome products IL-1β and IL-18 can bind to IL-1R and support the commitment of Th1 lymphocytes by inducing the release of IFN-γ. IFN-γ and IL-12 produced by tissue cells also promotes activation and differentiation of CD4^+^ lymphocytes to Th1 cells.

The effect of IL-18 on the Th1 type immune response also depends on IFN-γ release; however, the mechanisms are more complicated. Nakanishi et al. (2001) suggested that Th1 lineage differentiation requires combination of IL-18 and IL-12. The synergistic role of IL-12 for IL-18 stimulation of IFN-γ was confirmed by Bohn et al. (1998). In contrast, IL-18 without IL-12 co-stimulation will lead to differentiation of a Th2 immune response (Nakanishi et al., 2001). These data indicate that IL-12 could serve as a regulator of the Th1/Th2 switch. The role of IL-1β and IL-18 in differentiation of Th1 lymphocytes is summarized in Figure 3.

Inflammasome-linked activation of Th1 immune response was shown to have a pathogenic effect. Tye et al. (2018) have demonstrated that NLRP1 activation and release of IL-18 in combination with IFNγ could support destructive inflammation and decreased butyrate-producing Clostridiales in ulcerative colitis patients.

### Inflammasome and the Th2 Immune Response

Th2 differentiation is initiated by priming naïve Th0 cells *via* the TCR and the co-stimulatory molecule CD28 (Rulifson et al., 1997). This stimulation activates nuclear factor of activated T cells (NFAT), NFκb and activator protein-1 (AP-1) transcription activation factors (Li and Krammer, 2003), resulting in up regulation of interferon regulatory factor 4 (IRF4) expression (Gao et al., 2013). The cytokine milieu plays an important role in Th2 differentiation where IL-4 was shown to direct the polarization of lymphocytes (Hermanutz et al., 1978) by activation of the STAT6 pathway (Kaplan et al., 1996). Also, IL-4 can up regulate the master regulator GATA-binding protein 3 (GATA3) favoring the Th2 cell differentiation (Zhang et al., 1998; Lee et al., 2001). IL-2 can also activate STAT5, which is another regulator of Th2 cytokine production. These cytokines are transcriptionally activated by STAT5 through chromatin remodeling of the cytokine locus as well as maintaining GATA3 expression in differentiated Th2 cells (Zhu et al., 2003; Cote-Sierra et al., 2004). Activated Th2 cells produce IL-2 and IL-4 forming a powerful positive feedback loop maintaining the lymphocyte population.

The extent of an inflammasome activation can regulate the switch between Th1 and Th2 immune responses (Couper et al., 2008). Studies have shown that a prolong LPS activation of inflammasome triggers the release of IL-10 (Gurung et al., 2015b; Daley et al., 2017), a potent anti-inflammatory cytokine stimulating a Th2 immune response (Laouini et al., 2003). IL-10 can also modulate a Th2 response by preventing the overproduction of IL-4, IL-5, and IL-13 (Schandené et al., 1994; Joss et al., 2000; Wilson et al., 2007) cytokines which contribute to post-infection fibrosis (Nelson et al., 2000). The protective role of IL-10 could include inhibition of inflammasome signaling by preserving mitochondria integrity (Ip et al., 2017). Collected data suggest that the central role of IL-10 is protective by preventing the aggravated immune response and tissue damage induced by the inflammasome activation of a Th1 immune response (Gazzinelli et al., 1996; Hunter et al., 1997). However, IL-10 could also lead to chronic infection and failure to eliminate the pathogen (Anderson et al., 2007).

Another mechanism of Th2 activation includes the location of the inflammasome within the cell. In contrast to forming a cytosolic PRR, when the inflammasome is located in the nucleus, it could contribute to differentiation of lymphocytes into Th2 (Bruchard et al., 2015). It was shown that when in the nucleus, NLRP3 lacks inflammasome-forming potential, instead it interacts with the transcription factor IRF4 and binds to the promoter regions of *Il4* gene transactivating it. It appears that this intracellular localization of NLRP3 inflammasome is part of the naïve CD4^+^ T-cell differentiation, where Th1 had cytosolic, while Th2 had nuclear localization.

Activation of Th2 immune response by IL-18, a product of the inflammasome, was demonstrated by Sendler et al. (2020). It appears that a lack of IL-12 co-expression contributes to skewing the immune response toward a Th2 type. Another mechanism of IL-18 stimulation of the Th2 immune response was demonstrated using an animal model of *Leishmania* infection, a major infection which is characterized by increased serum level of IL-4 (Gurung et al., 2015a). It was found that GATA3 and cMAF expression was targeted by IL-18 in activated T cells, thereby biasing adaptive immunity toward a Th2 type. GATA is required for STAT5 activation of IL-2 dependent Th2 differentiation (Zhu et al., 2006) and directly activates the *Il4* promoter (Zheng and Flavell, 1997). Similar to GATA, cMAF can induce transcription of *Il4* (Zheng and Flavell, 1997). Additionally, absence of IL-12 was shown as contributing to IL-18 promotion of an Th2 immune response (Nakanishi et al., 2001).

A controversial role of IL-33 in regulation of Th2 immune response has been demonstrated (Schmitz et al., 2005; Cayrol and Girard, 2009). IL-33 is an IL-1-type cytokine, which is processed by Cas1, a product of inflammasome (Schmitz et al., 2005). It appears that based on the site of proteolytic digestion, Cas1 could activate or inhibit IL-33 (Schmitz et al., 2005; Cayrol and Girard, 2009). IL-33 is a cytokine promoting differentiation of naïve T cells to a Th2 phenotype and maintenance of the Th2 mediated immune response (Murakami-Satsutani et al., 2014). This cytokine binds to the suppressor of tumorigenicity 2 (ST2) receptor primarily expressed on Th2 cells and it was linked to their functions (Coyle et al., 1999; Townsend et al., 2000). Therefore, by Cas1 cleavage of IL-33, inflammasomes could promote or inhibit Th2 lymphocyte activation.

**Figure 4:**
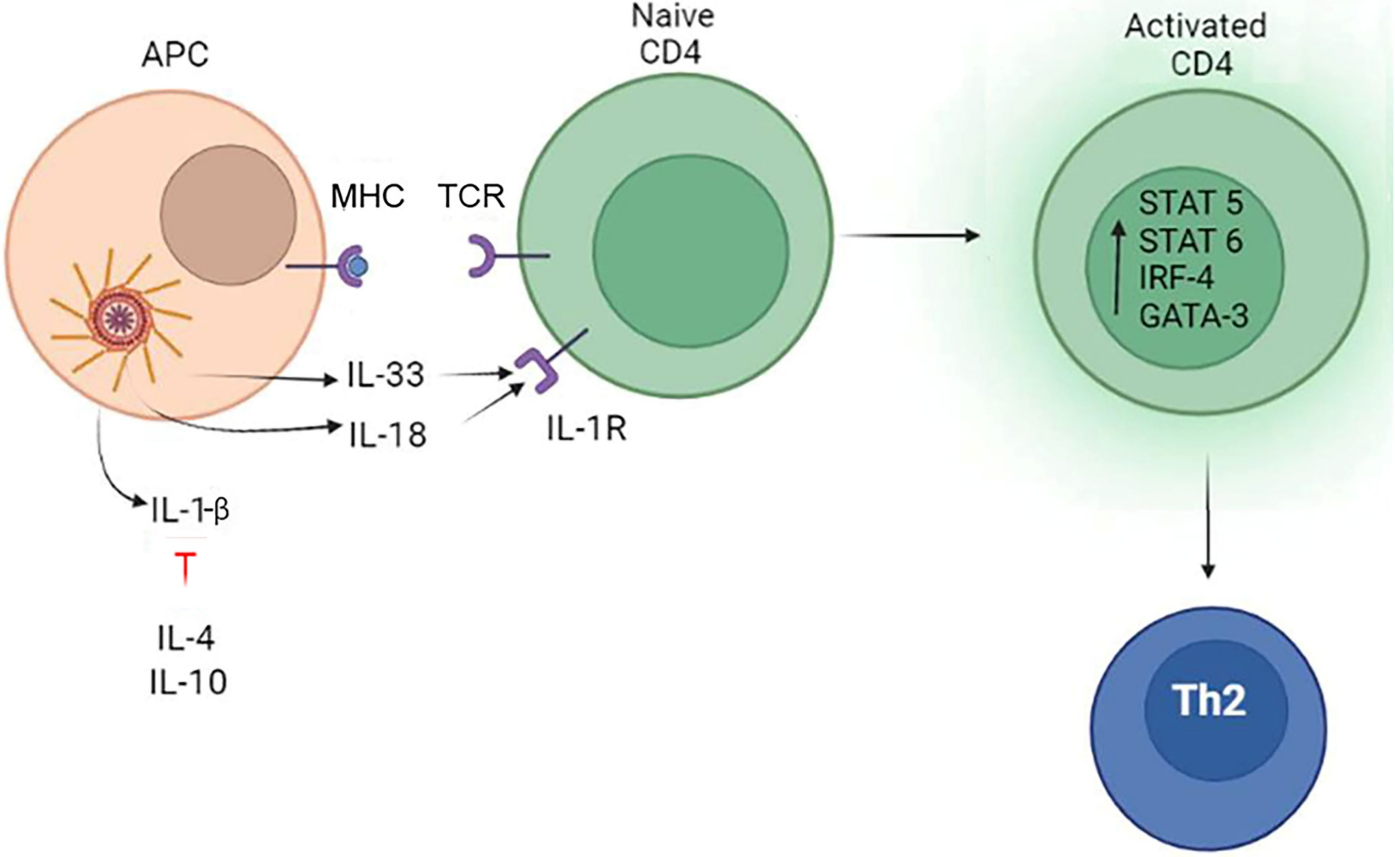
The role of the inflammasome in the differentiation of Th2 lymphocytes. The inflammasome produces IL-33 which can promote Th2 differentiation. Another inflammasome product, IL-18, can also promote Th2 lymphocytes development only in the absence of IL-12. Additionally, Th2 differentiation is promoted by tissue released IL-4 and IL-10, which blocks IL-1β activity.

Collectively, data suggest that presence of IL-4 and absence of IL-12 are required for IL-18 promotion of Th2 type immune response. Additionally, presence of active form of IL-33 could contribute to Th2 lymphocytes activation. The role of the inflammasome in differentiation of Th2 cells is summarized in Figure 4.

### Inflammasome and the Th17 Immune Response

Th17 cells differentiate from naive CD4^+^ cells after stimulation with TCR and subset of cytokines secreted by the APC (Bhaumik and Basu, 2017). Ivanov et al. (2006) have demonstrated that the retinoic acid receptor-related orphan receptor gamma-T (RORγt) is the key transcription factor regulating the differentiation of Th17 lymphocytes. It was shown that RORγt induces *Il17* transcription in naïve CD4^+^ T helper cells (Manel et al., 2008). The differentiation process is tightly regulated and includes differentiation (TGF-β and IL-6), proliferation (IL-21), and stabilization stages (IL-23; Luckheeram et al., 2012). TGF-β is the predominant signaling cytokine, which promotes Th17 differentiation by activating STAT3 (Qin et al., 2009). TFG-β can also activate RORyt, although this activation did not support the production of IL-17 (Manel et al., 2008). The expression of RORγt is highly unstable and requires IL-6 and STAT3 signaling (Korn et al., 2008). Also, the presence of IL-1β, IL-6, IL-21, or IL-23 was shown as required to stimulate IL-17 expression, which is done through the sustained activation of STAT3 (Yang et al., 2007; Zhou et al., 2007). The proliferation phase mainly involves IL-21 function, which synergizes with TGF-β to amplify Th17 proliferation (Korn et al., 2007; Nurieva et al., 2007). Stabilization of the Th17 population requires IL-23, which is produced upon RORyt induction (Manel et al., 2008). IL-23 receptor expression is upregulated by IL-6 and IL-21 (Zhou et al., 2007). The high level of IL-23 is required for maintenance of Th17 population (Langrish et al., 2005).

Th17 cells are characterized by the production of IL-17A, IL-17F, IL-21, and IL-22 cytokines (Spolski and Leonard, 2009). These lymphocytes protect against fungal and bacterial infection (Curtis and Way, 2009) by stimulating and recruiting neutrophils as well as triggering inflammation (Ye et al., 2001; Huang et al., 2004). It appears that Th17 lymphocyte activation requires IL-1β (Uchiyama et al., 2017), an inflammasome product, in combination with key cytokines IL-6 or TGF-β (Mangan et al., 2006; Zhou et al., 2007; Qin et al., 2009). IL-6 and TGFβ are the primary cytokines initiating Th17 development, while IL-1β was also shown capable of inducing IL-17 (Sutton et al., 2006). IL-6 is a critical differentiation factor for Th17 cells (Mangan et al., 2006) operating through the subsequent activation of STAT3 and RORγt in Th17 lymphocytes (Zhou et al., 2007). TGF-β could initiate expression of FoxP3 and RORγT during T helper cell priming (Fu et al., 2004; Zhang, 2018). However, it is co-stimulation with IL-6 that activates STAT3 mediated IL-17 expression and inhibits FoxP3 expression (Yang et al., 2011; O’Reilly et al., 2014; Latourte et al., 2017). This Th17 commitment of helper cells is promoted by IL-1β inhibition of SOCS3 (Qin et al., 2009; Basu et al., 2015), a negative regulator of STAT3 (Suzuki et al., 2001). Therefore, combined efforts of IL-1β, TGFβ, and IL-6 shift the balance from developing Treg to Th17 lymphocytes. In the absence of TGFβ, a combination of IL-1β, IL-6, and IL-23 could initiate development of a pathogenic Th1-like Th17 phenotype (Lee et al., 2012). This phenotype is believed to be pathogenic and characterized by production of IL-17, IFN-γ, IL-21, and IL-22 (Eisenbarth and Flavell, 2009). Additionally, it was demonstrated that IL-1β together with IL-21 and IL-23 could guide the Th17 commitment (Bhaumik and Basu, 2017).

IL-1β acts through binging to IL-1R which is indispensable for the early differentiation of Th17 (Sutton et al., 2006). Additionally, IL-1β promotes Th17 differentiation by synergizing with IL-6 and upregulating the master transcription factors, such as STAT3, IRF4, and RORγt (Basu et al., 2015). IL-1/IL-1R axis facilitates the binding of STAT3 and NF-κB to the cis-regulatory elements leading to enhanced transcription of IL-17A and IL-17F (Whitley et al., 2018). Additionally, IL-1β signaling regulates the expression of a transcription factor Bhlhe40, which was found in cells producing IL-17 (Lin et al., 2016).

**Figure 5:**
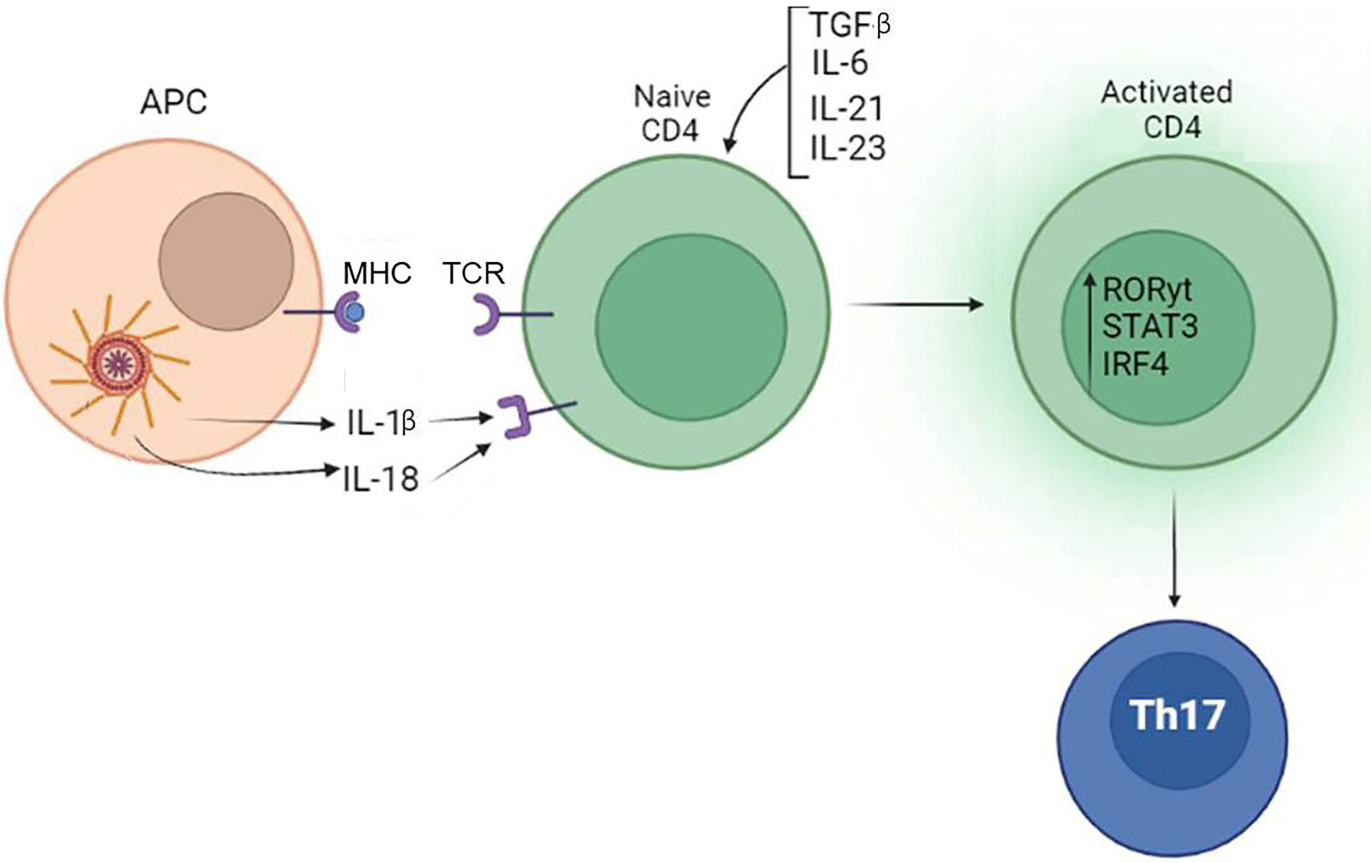
Inflammasome contribute to differentiation of Th17. IL-1β produced by the inflammasome, when combined with TGF-β, IL-6, IL-21, and IL-23 produced by tissue cells, can promote the differentiation of Th17 lymphocytes.

These data suggest that the inflammasome product, IL-1β, could contribute to Th17 induction and commitment, which requires co-stimulation with IL-6, TGF-β, IL-21, and IL-23. The role of inflammasome in Th17 differentiation is summarized in Figure 5.

## Conclusion

Inflammasomes, a cytosolic PRR, are integral components of an innate immune response producing key pro-inflammatory cytokines. However, inflammasome function is not restricted to the induction of inflammation as growing evidence suggests that they also can impact and shape specific adaptive immunity.

**Figure 6:**
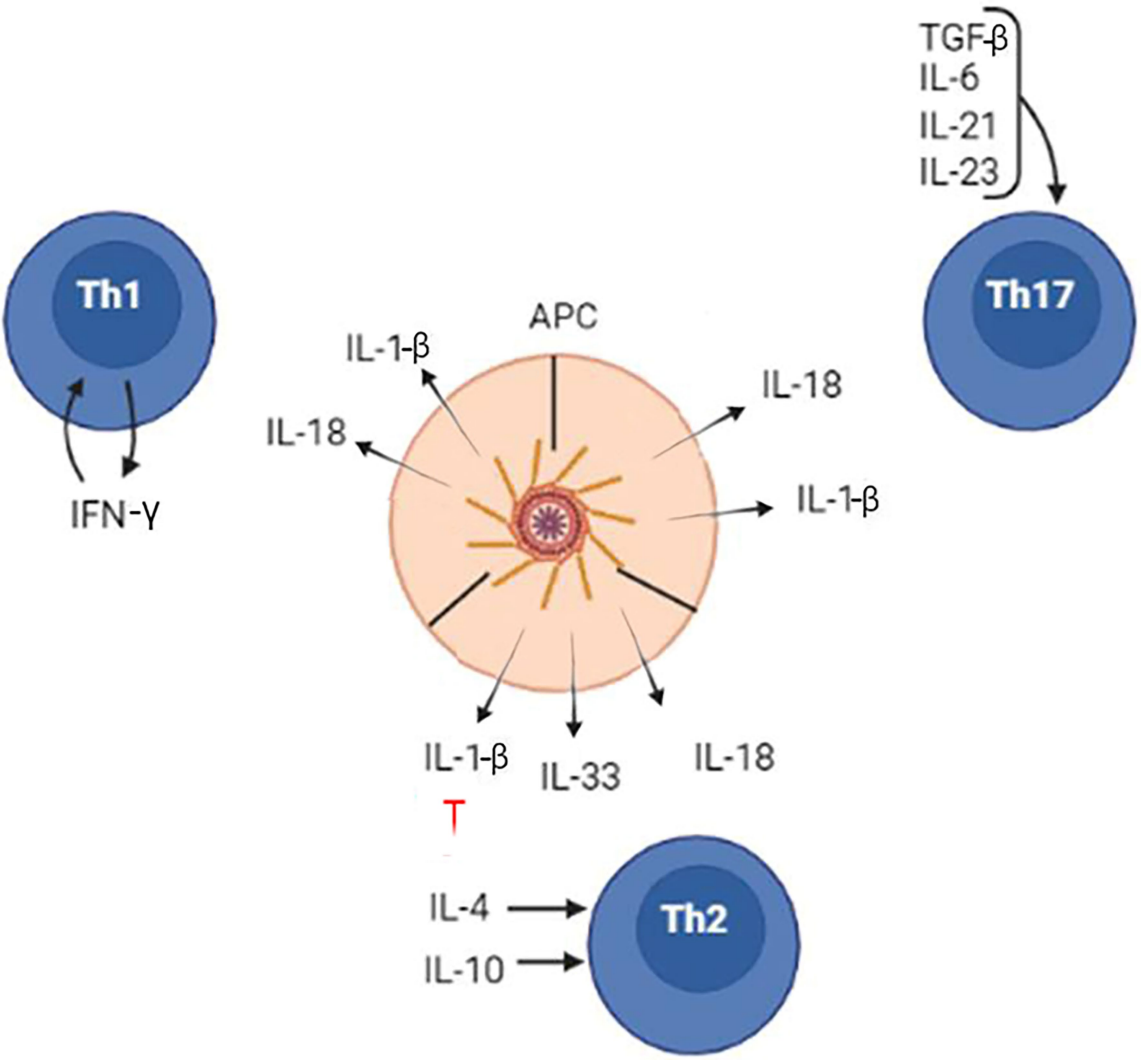
The inflammasome role in the differentiation of naïve CD4 lymphocytes to Th1, Th2, and Th17 cells. Inflammasome products, IL-1β, IL-18, and IL-33, contribute to differentiation of Th1, Th2, and Th17 lymphocytes. However, the lymphocyte commitments and differentiation require additional cytokines, which are released by local tissues.

Shaping the immune response by inflammasomes is accomplished mainly by production of two cytokines, IL-1β and IL-18. These cytokines contribute to the development of Th1, Th2, and Th17 lymphocytes and require co-stimulation (Figure 6). When inflammasome product IL-1β is combined with IFN-γ, naïve T cells will differentiate to a Th1 subset. Also, Th1 differentiation will occur when IL-18, another inflammasome product, is combined with IL-12. In contrast, only IL-18 in the absence of IL-12 could initiate naïve T lymphocytes differentiation toward a Th2 type. Inhibition of inflammasomes by IL-10 could also promote Th2 differentiation. Naïve T-cell differentiation and commitment to Th17 is managed by release of IL-1β and its cooperation with IL-6 or TGFβ.

These co-stimulatory cytokines required for Th lineage commitment are released by the tissue cells and other leukocytes. The release of these cytokines is initiated by inflammasome products, such as IL-1β and IL-18. However, the type of co-stimulatory cytokine(s) would depend on the tissue type and nature of the stimulus.

In conclusion, we have detailed how the inflammasome is an essential part of how the immune system responds to a variety of insults, and how it bridges both the innate and adaptive arms of the immune system.

## Author Contributions

EM and SK: conceptualization. AR: funding acquisition. EM: supervision. EM, RU, and SK: writing—original draft. SK, EM, RU, and AR: writing—review and editing. All authors contributed to the article and approved the submitted version.

## Funding

This work was funded, in part, by the Russian Government Program of Competitive Growth of Kazan Federal University. Also, this work was supported by the Kazan Federal University Strategic Academic Leadership Program.

## Conflict of Interest

The authors declare that the research was conducted in the absence of any commercial or financial relationships that could be construed as a potential conflict of interest.

## Publisher’s Note

All claims expressed in this article are solely those of the authors and do not necessarily represent those of their affiliated organizations, or those of the publisher, the editors and the reviewers. Any product that may be evaluated in this article, or claim that may be made by its manufacturer, is not guaranteed or endorsed by the publisher.

## Glossary

**Table:** 

Abbreviation	Definition
AIM2	Absent in melanoma 2
ALRs	AIM2 like receptors
APCs	Antigen-presenting cells
ASC	Apoptosis-associated speck-like
APAF1	Apoptosis protease-activating factor 1
Cas1	Caspase-1
CARD	Caspase activation and recruitment domain
CTLs	Cytotoxic T lymphocytes
GSDMD	Gasdermin D
HIN	Hematopoietic interferon-inducible nuclear
IRF	Interferon regulatory family
ICE	IL-1β-converting enzyme
IPAF	ICE protease-activating factor
LRR	Leucine-rich repeat
LPS	Lipopolysaccharide
MDP	Muramyl dipeptide
NK	Natural killer
NAIP	Neuronal apoptosis inhibitory protein
NLRC	NLR family CARD domain-containing protein
NLRP	NLR family Pyrin domain
NLRs	NOD-like receptors
NFAT	Nuclear factor of activated T cells
NODs	Nucleotide-binding and oligomerization domain
P2X7	P2X purinoceptor 7
PAMPs	Pathogen-associated molecular patterns
PRRs	Pattern recognition receptors
PYD	Pyrin domain
RORγt	Retinoic acid receptor-related orphan receptor gamma-T
RLR	RIG-1-like receptors
STAT1	Signal transducer and activator of transcription 1
TCR	T-Cell receptor
Th1	T Helper 1
TLRs	Toll-like receptors
T3SS	Type 3 secretion system
